# Management of Intraoperative Miosis during Pediatric Cataract Surgery using Healon 5

**DOI:** 10.4103/0974-9233.75888

**Published:** 2011

**Authors:** Vishal Jhanji, Namrata Sharma, Rasik B. Vajpayee

**Affiliations:** 1 Department of Ophthalmology and Visual Sciences, The Chinese University of Hong Kong, Hong Kong; 2 Department of Ophthalmology, Royal Victorian Eye and Ear Hospital, Centre for Eye Research Australia, University of Melbourne, Victoria, Australia; 3 3Department of Ophthalmology, Dr Rajendra Prasad Centre for Ophthalmic Sciences, All India Institute of Medical Sciences, New Delhi, India

**Keywords:** Intraoperative Miosis, Pediatric Cataract, Viscoelastic, Viscomydriasis

## Abstract

**Purpose:**

We describe a technique for achieving pupillary dilatation in order to manage and counteract intraoperative miosis during pediatric cataract surgery using viscoadaptive viscoelastic (sodium hyaluronate 2.3%).

**Materials and Methods:**

The technique of viscomydriasis was used in six eyes with pediatric cataracts with intraoperative pupillary miosis.

**Results:**

Pupillary dilatation was achieved and maintained in all eyes throughout cataract surgery. All the surgical steps including anterior and posterior capsulorrhexis and aspiration were performed successfully.

**Conclusions:**

Viscomydriasis is a simple and effective technique for the management of intraoperative pupillary miosis during cataract surgery in pediatric eyes.

## INTRODUCTION

Surgical management of cataracts in infants is one of the most challenging situations in ophthalmic cataract surgery. 1 Achieving adequate mydriasis is generally difficult in infants particularly in children less than 6 months of age due to iris hypoplasia and associated conditions such as rubella. 2 We have encountered some cases of intraoperative miosis despite adequate preoperative pupillary dilatation with pharmacological agents. In this report, we describe a technique for managing intraoperative miosis in infants with cataracts using viscoadaptive ophthalmic viscosurgical devices (OVDs).

## MATERIALS AND METHODS

Six eyes of three children are documented in this study. This study was approved by the Institutional Review Board of the Hospital and was in accordance with the ethical standards laid down in the Declaration of Helsinki. An informed consent was obtained from the parents of all the patients.

### Surgical technique

**Figure 1:**
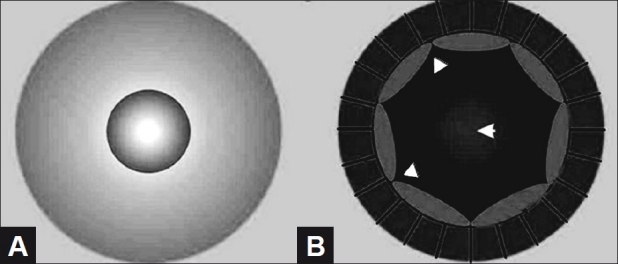
Schematic showing intraoperative miosis (A) and viscomydriasis (B); (arrowheads depict Healon 5)

The technique of viscomydriasis was used in six eyes with pediatric cataracts with intraoperative pupillary miosis. In all these eyes, cataract surgery was performed under general anesthesia. Adequate preoperative dilatation was achieved using atropine sulfate 1% eye ointment (Cipla, Mumbai, India). The surgical technique involved creation of a 2.75 mm biplanar limbal incision made on the superior limbus with a keratome (Alcon Labs, Fort Worth, TX). Trypan blue dye (0.06%) (Shah and Shah, Calcutta, India) was used to stain the anterior lens capsule under air bubble. The anterior chamber was filled with 1.4% sodium hyaluronate. The anterior continuous curvilinear capsulorrhexis involved the creation of an initial capsule flap with a cystotome followed by completion of the capsulorrhexis with Uttrata forceps. Hydrodissection and hydrodelineation were performed after aspiration of lens material with the Protege phacoemulsification machine (Storz Protégé, Bausch and Lomb Inc., Rochester, NY). When intraoperative miosis was noted, intracameral adrenaline (preservative free, 0.1 mL 1:1000) was used as an initial step to dilate the pupil. In the event of failure of dilation of pupil after intracameral adrenaline administration, boluses of 2.3% sodium hyaluronate (Healon 5, AMO Inc., CA, USA) were injected around the pupillary margin in order to mechanically dilate the pupil to a size of 6-7 mm.[Figure 1] Intraoperatively, care was taken to perform the lens aspiration under the capsulorrhexis margin avoiding the pupillary margin. In the event of failure to achieve viscomydriasis, small sphincterotomies were planned.

After complete aspiration of cortical lens material a small amount of Healon 5 was re-injected and a posterior capsulorrhexis was performed using Uttrata’s forceps followed by anterior vitrectomy. Subsequently, irrigation and aspiration was performed meticulously in order to remove the Healon 5 from the anterior chamber. Extra care was taken to avoid directing the flow of the irrigation cannula toward the posterior capsulorrhexis. At the end of the surgery the limbal wound was closed with a single 10-0 monofilament nylon suture.

## RESULTS

The technique of viscomydriasis was used in six eyes of three children (mean age, 1.4±0.41 years) with congenital cataracts. Intraoperative miosis was encountered during trypan blue staining of the anterior capsule in two eyes and during hydrodissection in four eyes. Intracameral adrenaline failed to achieve optimal pupillary dilatation in any of the eyes. Adequate pupillary dilatation could be achieved in all eyes after injection of 2.3% sodium hyaluronate inside the pupillary margin. In four eyes a repeat injection of viscoelastic was required due to inadvertent aspiration of the viscoelastic and recurrence of intraoperative miosis during aspiration of lens material. None of the eyes required a pupillary sphincterotomy. In all eyes, the entire surgical procedure including anterior and posterior capsulorrhexis and lens material aspiration could be performed optimally. All eyes were left aphakic at the end of the surgery. No other intraoperative complications were observed. Examination under anesthesia on day 3 showed clear corneas and normal intraocular pressure in all cases.

## DISCUSSION

Intraoperative miosis during cataract surgery is a well-known entity in adults. 3 Use of diclofenac sodium and flurbiprofen preoperatively, addition of epinephrine in the irrigating solution, use of intracameral adrenaline, iris hooks or multiple sphincterotomies have been found to be effective in maintaining intraoperative mydriasis during cataract surgery. 4 In spite of achieving a good preoperative pupillary dilatation, we observed intraoperative miosis in some of our cases of pediatric cataract surgery, making it difficult to perform the surgery. In the newborn or infant, the pupils are miotic and dilate very poorly. At birth, the dilator muscle is poorly developed, which may explain the difficulty in obtaining adequate mydriasis in infants. 5

Viscoelastics have been used in the past to achieve intraoperative pupillary dilatation. 6,7 Healon 5 is a high molecular weight mass fraction of sodium hyaluronate. It has high viscosity and elasticity when exposed to low and high shear rates. The pseudodispersive properties of Healon 5 allow it to remain inside the eye at low flow rates. This helps to create as well as preserve space within the anterior chamber while the lower viscosity dispersive agents are retained in the eye, thereby achieving the desired compartmentalization. Jeng *et al*. compared the efficacy of different viscoelastics for completion of continuous capsulorrhexis in pediatric cataracts and found Healon 5 to be superior to other commonly used viscoelastics. 8 Similar results have been reported by Gibbon *et al*. in children younger than 5 years. 9 In our cases intraoperative miosis was encountered during trypan blue staining and hydrodissection maneuvers, possibly due to the decompression of the eye during these surgical steps. We injected Healon 5 as soon as intraoperative miosis was noted. A thick bolus of Healon 5 does not easily get expelled and can keep the pupil mechanically dilated during surgery. If specific attempts are not made to aspirate this viscoelastic, it can remain inside the eye for a long duration to keep the iris stretched. It also prevents any trauma to the iris thereby making this technique superior to the mechanical stretching of the iris.

In our cases Healon 5 injection enabled the surgeon to create as well as to maintain working space inside the eye while providing adequate pupillary dilatation required for safe surgery. Furthermore, a repeat injection can be easily performed in case some of the viscoelastic is aspirated as occurred in 4 eyes in this series. We believe that our technique is a simple and effective technique to counteract intraoperative miosis during pediatric cataract surgery.
